# Epstein-Barr Virus-Associated Smooth Muscle Tumor and Its Correlation With CD4 Levels in a Patient With HIV Infection

**DOI:** 10.3389/fcimb.2022.725342

**Published:** 2022-01-24

**Authors:** Yoon Bin Chong, Po-Liang Lu, Yu-Chun Ma, Hsin-Ling Yin, Chih-Hui Chang

**Affiliations:** ^1^ Division of Neurosurgery, Department of Surgery, Kaohsiung Medical University Hospital, Kaohsiung Medical University, Kaohsiung, Taiwan; ^2^ School of Medicine for Post-Baccalaureate, College of Medicine, Kaohsiung Medical University, Kaohsiung, Taiwan; ^3^ Division of Infectious Disease, Department of Internal Medicine, Kaohsiung Medical University Hospital, Kaohsiung Medical University, Kaohsiung, Taiwan; ^4^ Center for Liquid Biopsy and Cohort Research, Kaohsiung Medical University, Kaohsiung, Taiwan; ^5^ Department of Pathology, Kaohsiung Medical University Hospital, Kaohsiung Medical University, Kaohsiung, Taiwan

**Keywords:** HAART, IRIS - immune reconstitution inflammatory syndrome, CD4, HIV, case report

## Abstract

Epstein-Barr virus-associated smooth muscle tumor (EBV-SMT) is a rare tumor found in immunocompromised patients, and its treatment is not well-established. A role for antiretroviral therapy in human immunodeficiency virus (HIV)-related EBV-SMT has been proposed; however, the relevance of tumor size, CD4 levels, and immune reconstitution inflammatory syndrome (IRIS) has not been previously reported. We present the first case, to our knowledge, of a tumor that shrank in association with elevated CD4 counts. IRIS occurred in this case following antiretroviral therapy. This finding highlights the importance of the immune response in HIV-related EBV-SMT.

## Introduction

The Epstein-Barr virus (EBV) is a human herpesvirus. Its latent form is very common, and almost 95% of the human population has been infected (Yan et al., 2018). However, in immunocompromised patients, such as those infected with the human immunodeficiency virus (HIV), under iatrogenic immunosuppression after transplantation, or with congenital immunodeficiency, the risk of EBV reactivation is much higher. It is well known that EBV causes tumorigenesis in Burkitt lymphoma, post-transplantation lymphoproliferative disease, and nasopharyngeal carcinoma. EBV is estimated to cause approximately 200,000 new tumors per year (Issarachaikul et al., 2014; Shannon-Lowe and Rickinson, 2019). Annually, 1%–6% of HIV-infected individuals develop lymphoma (Bibas and Antinori, 2009). Although the connection between EBV and EBV-associated smooth muscle tumor (EBV-SMT) is well-established, the underlying pathophysiology is not well-understood. Due to the rarity of EBV-SMT, the diagnosis and treatment of this tumor were unfamiliar to us (Chiu et al., 2018). Malignant smooth muscle tumors were first reported in the 1970s in immunosuppressed patients (Pritzker et al., 1970). In the 1990s, the association between EBV and malignant smooth muscle tumors was identified and later defined as EBV-SMT (Liebowitz, 1995; McClain et al., 1995). The tumors are mainly categorized into three groups according to the origin of immune deficiency as follows: 1. HIV-related EBV-SMT, 2. post-transplant-related EBV-SMT, and 3. congenital immunodeficiency-related EBV-SMT. EBV-SMT is extremely rare; no exact prevalence is reported, but it is estimated to be <2 cases per million (Deyrup et al., 2006). In HIV-related EBV-SMT, the tumor is predisposed to occur in the brain, spine, liver, and adrenal glands. Most patients have multifocal tumors at the time of diagnosis, which are not considered as metastases but rather as multiple independent tumor occurrences (Lee et al., 1995). Most published studies focus on the surgical resection of this tumor for diagnosis and treatment; however, since 26%–53% of EBV-SMTs present as a multifocal tumor at the time of diagnosis, it is sometimes impossible to resect the tumors completely (Pritzker et al., 1970; Issarachaikul et al., 2014). Other studies suggested additional radiotherapy for unresected or multifocal tumors. Chemotherapy has been suggested as a treatment option; however, a poor response has been reported. Mammalian target of rapamycin (mTOR) inhibitors have yielded promising results in post-transplant-related EBV-SMT (Hussein et al., 2014), and some congenital immunodeficiency-associated EBV-SMTs showed tumor control after bone marrow transplantation (Magg et al., 2018). In HIV-related EBV-SMT, highly active antiretroviral therapy (HAART) is indicated because of the high risk of death due to infection rather than tumor progression. Suankratay et al. reported tumor control with HAART, but all patients underwent surgery, and the relationship with CD4 levels was not shown (Suankratay et al., 2005). Based on their report, it is tempting to hypothesize that EBV-SMT is similar to Kaposi sarcoma in that the tumor can be controlled after an increase in CD4 count. Here, we report a case with EBV-SMT; the tumor shrank without surgery in close association with the CD4 count, and the phenomenon of immune reconstitution inflammatory syndrome (IRIS) was observed.

## Case Presentation

**Figure 1:**
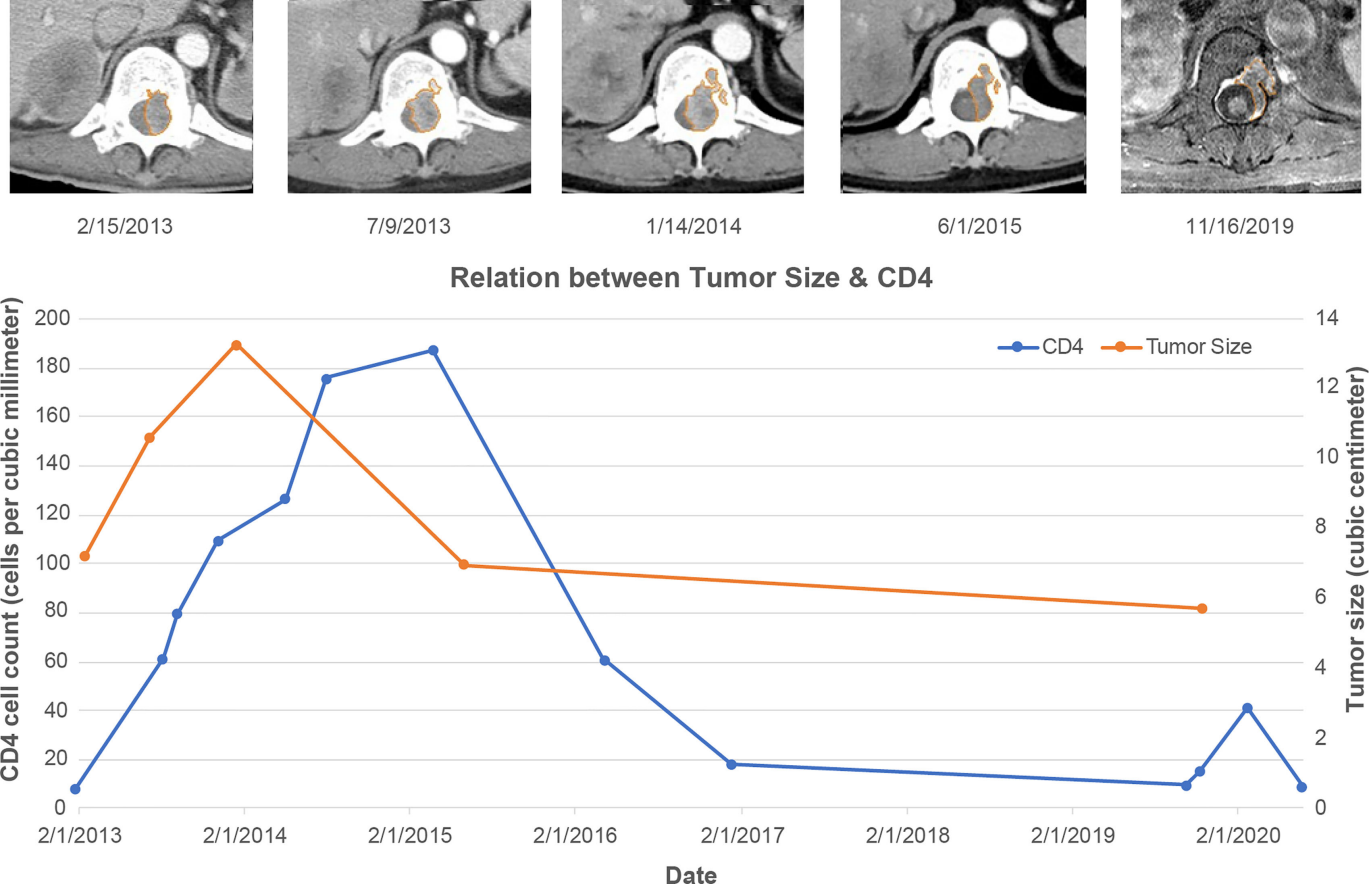
Relationship of T11 Tumor Size with CD4 Count. The size of the tumor slowly increases until it peaks at 01/14/2014, despite elevated CD4 levels during the 2013 to 2015 period. The tumor then begins to decrease in size. Upper row: Abdominal computed tomography and magnetic resonance imaging (MRI) at different time points showing an Epstein-Barr virus-associated smooth muscle tumor at the T11 level, and the tumor size is highlighted with an orange line. The MRI of the tumor at the right end shows that the tumor has no mass effect on the spinal cord.

A 38-year-old male patient with a 12-year history of HIV and hepatitis B co-infection received initial antiretroviral therapy (ART) with lamivudine (3TC 150 mg Q12H; GlaxoSmithKline, Brentford, UK), stavudine (Zerit 40 mg Q12H; Emcure Pharmaceuticals, Pune, India), and efavirenz (Stocrit 600 mg QD; Merck, Kenilworth, NJ). He was not compliant with medical care and was lost to follow-up for 2 months. Subsequently, he experienced an episode of general malaise and was diagnosed with neutropenic fever due to a gluteal abscess. Despite antibiotic treatment at admission, progression to septic shock occurred. He had a CD4 count of 6 cells/µL and an HIV viral load of 18,251 copies/mL. We arranged an enhanced abdominal computed tomography (CT) scan to exclude other intra-abdominal infections. Bilateral adrenal gland tumors were noted; the right tumor had a volume of 40 cm^3^, and the left tumor had a volume of 7 cm^3^. Both tumors presented as hypovascular solid tumors with low central densities on CT. Furthermore, an incidental T11 paraspinal tumor was observed on abdominal CT. Enhanced magnetic resonance imaging (MRI) of the thoracolumbar spine revealed a T11 extradural tumor. This tumor had a volume of approximately 7 cm^3^ and was of extradural origin with good enhancement. Intermittent numbness of the legs was the only neurological symptom at that time; the patient refused surgery but agreed to a CT-guided biopsy of one of the adrenal tumors. The pathology results revealed leiomyoma. With ART, the patient’s CD4 count increased slowly; however, the size of the T11 paraspinal tumor also increased slowly and reached a maximum size of 13 cm^3^ while the CD4 count remained at approximately 108 cells/µL (**Figure 1**).

**Figure 2:**
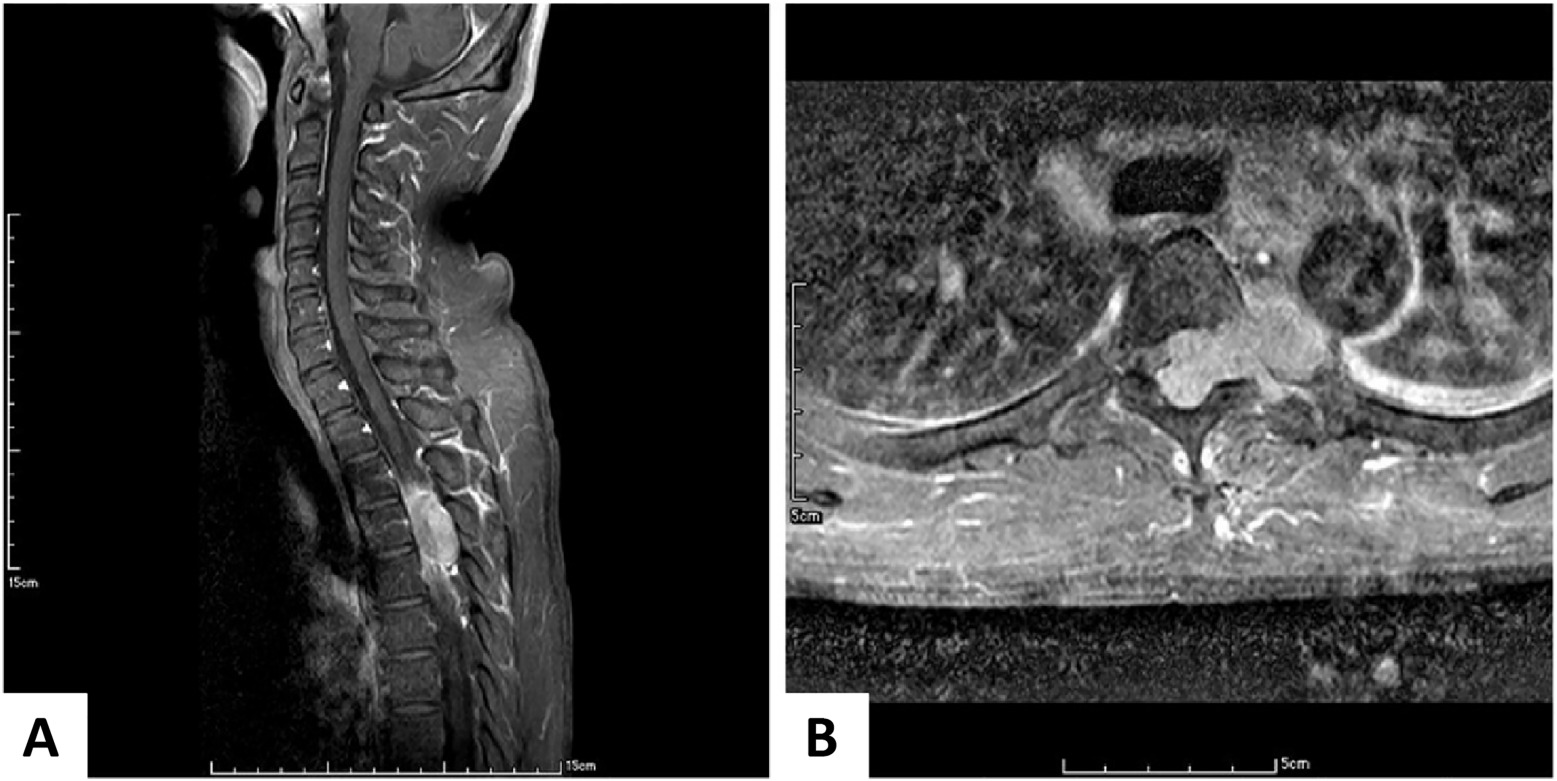
Enhanced Magnetic Resonance Imaging of the Cervicothoracic Spine. **(A)** In the sagittal view, a large enhancing epidural tumor arising from T4 compresses the spinal cord. **(B)** In the axial view, this tumor arises from the left neural foramen and pushes the spinal cord to the right. Similarly, the tumor involves the aorta.

Two years later, the fifth follow-up CT revealed that the T11 tumor shrank in size to 6 cm^3^ when the CD4 count reached 186 cells/µL, which was the highest value during his treatment. His numbness symptoms had improved at this point. However, he was lost to follow-up from January 2017 until October 2019, and he did not take any medication or undergo diagnostic imaging during the period from 2017 to late 2019. When he presented after almost 3 years, his CD4 count had dropped to 8 cells/µL, while the tumor diameter remained at 5.6 cm^3^. ART was initiated at this time. He sought medical help for neutropenic fever and progressive paraplegia that had developed in the 1-month period after ART initiation. An MRI of the cervicothoracic spine revealed a large T4 extradural tumor with spinal cord compression (**Figure 2**).

**Figure 3:**
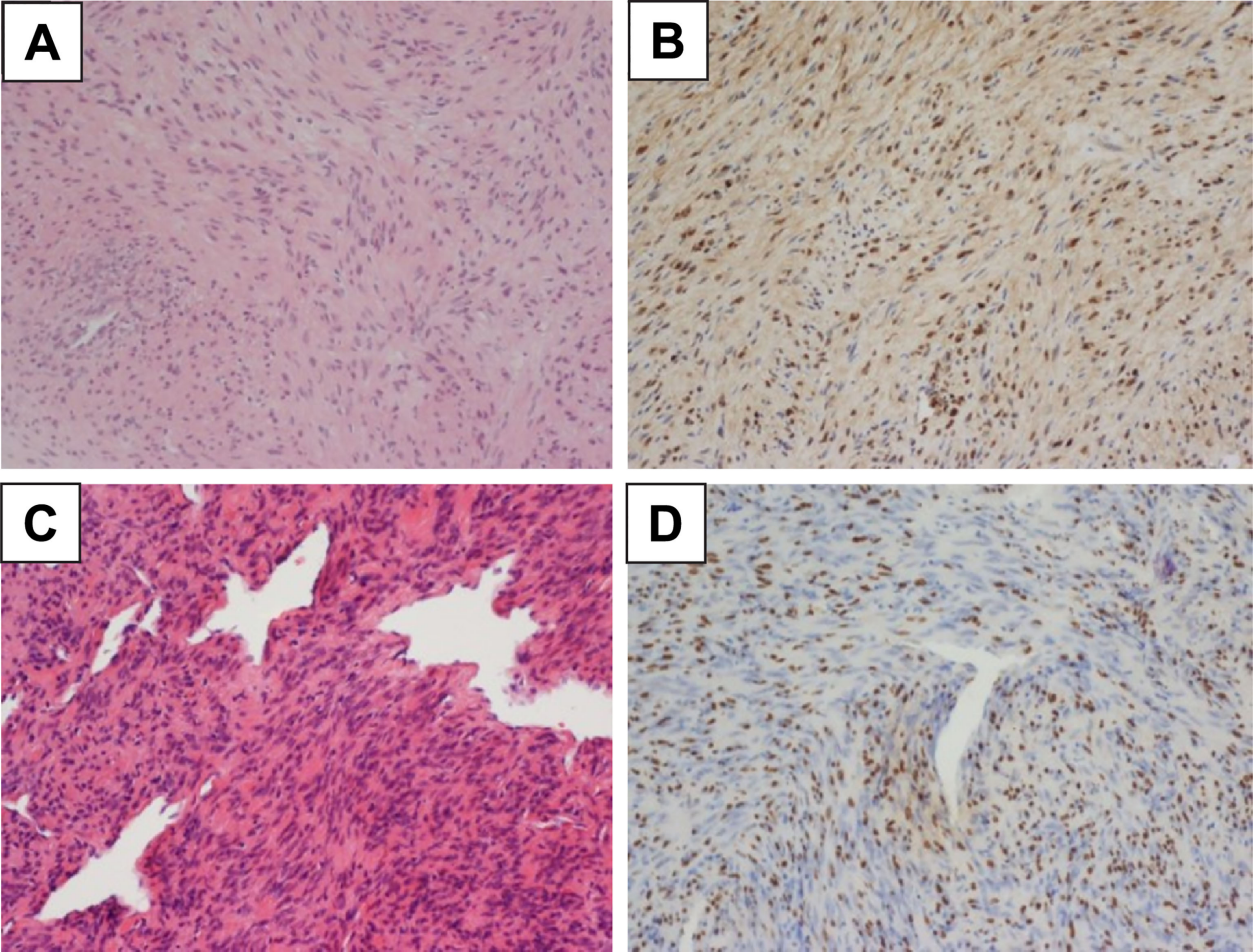
Microscopic Examination of the Histopathological Specimen. **(A)** The bilateral adrenal tumors are composed of intersecting fascicles of spindle cells, with abundant eosinophilic cytoplasm and elongated nuclei and a hemangiopericytoma-like pattern. (200X HE stain) **(B)** Epstein-Barr virus-encoded RNA is detected by *in situ* hybridization in the adrenal tumor. (200X) **(C)** The extradural tumor is also composed of intersecting fascicles of spindle cells and branching vessels. Morphological features are similar to those of the adrenal tumor. (200X HE stain) **(D)** Epstein-Barr virus-encoded RNA is additionally detected by *in situ* hybridization in the extradural tumor (200X). The bilateral adrenal tumors are composed of intersecting fascicles of spindle cells, with abundant eosinophilic cytoplasm and elongated nuclei and a hemangiopericytoma-like pattern. (200X HE stain).

Surgery was performed for decompression, and the frozen-section pathology suggested schwannoma. After consultation with the pathologists, the final diagnosis was an EBV-SMT (**Figure 3**). The histology of the specimen was similar to that of a normal smooth muscle tumor with intersecting fascicles of spindle cells and branching vessels. The tumor stained positive for EBV-encoded small RNAs, which is the gold standard for diagnosing EBV-SMT. Subsequently, we arranged EBV-encoded small RNAs for the adrenal gland tumor biopsy and established EBV-SMT diagnosis (**Figure 3**).

Radiation therapy was arranged for the remaining T4 tumor. The patient later underwent rehabilitation and became ambulatory with a walker. The tumor remained stable for 1 year after surgery and remained under follow-up.

## Discussion

Our case report reminds physicians to perform further histological studies when a smooth muscle tumor is found in an unusual location. In our case, we did not consider an EBV-SMT diagnosis when we detected the leiomyoma of the adrenal gland, and we only arranged regular follow-up imaging. The final diagnosis became apparent when the patient presented with paraplegia due to spinal cord compression caused by the thoracic tumor, and the pathology findings revealed that the smooth muscle tumor was an EBV-SMT (Chiu et al., 2018).

The pathology of an EBV-SMT is similar to that of other smooth muscle tumors. Some studies described that EBV-SMT tissue samples contained lymphocytic cells in addition to the usual fusiform and spindle-shaped cells; in our case, the morphology appears to be that of a leiomyoma (Deyrup et al., 2006; Hussein et al., 2014). Unlike in leiomyosarcoma, the mitotic rate of an EBV-SMT is not relevant to the patient’s survival; thus, the term “sarcoma” is not used. The gold standard for diagnosing EBV-SMT is EBV-encoded small RNA *in situ* hybridization because immunohistochemistry alone has a rather low sensitivity (Magg et al., 2018). It is particularly important to establish an algorithm for the pathological diagnosis of spindle cell tumors in HIV patients, as other differential diagnoses include Kaposi sarcoma, myopericytoma, gastrointestinal stromal tumor, and mycobacterial spindle cell pseudotumor (Zhou et al., 2020).

The prognosis of HIV-related EBV-SMT is poorer than that of post-transplant EBV-SMT, with a mortality rate of 40% compared to 30% for post-transplant EBV-SMT. Most patients die of opportunistic infection rather than tumor progression (Calafiore et al., 2020). The pathophysiology of the tumor is not well-understood. It is postulated that EBV enters mesenchymal cells *via* CD21 since most HIV-related EBV-SMTs stain positive for CD21, whereas post-transplant-related EBV-SMTs are usually CD21-negative (Jenson et al., 1999). Similarly, the fusion between human embryonic fibroblast cells and EBV-infected lymphoblastoid cells has been described. These studies demonstrate that the exact entry mechanism is unknown (Deyrup, 2008). Currently, the molecular tumorigenesis of EBV-SMT is suspected to be related to mTOR pathways since activation of mTOR signaling and v-*myc* (the oncogene of viral myelocytomatosis) cause the proliferation of EBV-SMTs (Hussein et al., 2014; Magg et al., 2018).

The treatment of EBV-SMT mainly focuses on surgical resection because it is required for pathologic confirmation. However, due to the high probability of having multiple tumors, it is not always possible to completely resect all tumors, and radiation therapy is sometimes added to treat incompletely resected tumors. EBV-SMT is chemotherapy-resistant; thus, the result is less favorable (Deyrup et al., 2006; Calafiore et al., 2020; Reddy et al., 2020). Due to mTOR activation in post-transplant-associated EBV-SMT, some studies have reported the successful use of sirolimus for tumor control while simultaneously preventing transplant rejection (Tan et al., 2013). In congenital immunodeficiency-related EBV-SMT, tumors have been successfully treated with bone marrow transplantation (Yonkof et al., 2020). Since most patients with EBV-SMT die of opportunistic infection rather than the tumor, immune reconstitution is the most important treatment. In patients with HIV-related EBV-SMT, HAART is necessary to prevent opportunistic infections, and due to the promising treatment effect in congenital immunodeficiency- and post-transplant-related EBV-SMTs, it also suggested that secondary increased CD4 counts can assist in the treatment of HIV-related EBV-SMTs. Recent studies state that increased CD4 levels after HAART can lead to disease stabilization or regression; however, all described EBV-SMT cases involved prior surgery. In contrast, the tumor in our case regressed to a minimal size after increased CD4 counts without surgery, although it remained latent. The pathophysiology of this behavior is not well-understood; however, it has been postulated that EBV-SMT behaves like Kaposi sarcoma. In a study examining HIV-related Kaposi sarcomas that were combination ART-naïve, approximately 80% of patients experienced tumor regression after treatment initiation (Bower et al., 2009). Although the exact mechanism of the immune response on tumor regression in EBV-SMT and Kaposi sarcoma is unknown, it has been hypothesized that cytotoxic immune cells are responsible for the observed effect. In EBV-SMT, EBV is in its type III latent form, which expresses EBNA1, EBNA2, LMP1, and LMP2A, as demonstrated by Rogatsch et al. (2000). This finding clarifies the mechanism of immune modulation in EBV-SMT. EBNA2 is highly recognizable by cytotoxic T cells, and immune reconstitution increases the capability of T cells to kill tumor cells, ultimately leading to tumor shrinkage over time (Creager et al., 1998). Interestingly, despite the increased CD4 count in our case, the tumor size increased slowly for 1 year and subsequently began to shrink. This behavior suggests that this case presented an IRIS-like phenomenon. As in IRIS-related Kaposi sarcoma, there is a temporal connection between CD4 count and progression of the Kaposi sarcoma. Before the immune system is reestablished, there are two phases: redistribution and repopulation. The redistribution phase takes several months and is related to increased numbers of memory T cells. It is followed by the repopulation phase, in which naïve T cells are generated, and the immune system is established (Bower et al., 2005). Since IRIS in EBV-SMT has not been previously reported, there are no criteria for diagnosing this phenomenon. We adopted the criteria of the diagnosis of IRIS proposed by French et al. for Kaposi sarcoma (French et al., 2004). According to this proposal, an IRIS phenomenon can be diagnosed by the enlargement of the tumor after initiating HAART, at least an 18-fold increase in CD4 count during this period, and shrinkage of the tumor without any radiation therapy or chemotherapy. This is the first reported case of EBV-SMT shrinkage following only ART with observed IRIS phenomenon, and it suggests that ART should remain the mainstay of EBV-SMT treatment.

This study has some limitations. First, we did not conduct a direct biopsy of the T11 tumor, and we could only perform EBV-encoded small RNA *in situ* hybridization on the adrenal tumor to prove the presence of an active EBV-SMT in the patient. Additionally, we could only retrospectively hypothesize that the T11 tumor was EBV-SMT. We decided to study the relationship between the CD4 count and the T11 tumor instead of the adrenal tumor because we did not arrange abdominal CT after 2016 and adrenal tumor size could not be evaluated very well in the spinal MRI. Second, we calculated the tumor size using the CT and MRI areas; however, due to the different planes of an axial view obtained using the two modalities, a direct comparison was not possible because of the unavailability of CT images after 2016. Third, between 2016 to 2020, the tumor size remained around 5.6–6cm^3^ despite the drop in CD4count below 60 cells/mm^3^, which according to our hypothesis, should have made the tumor size larger. We speculated that during this period, the tumor size should initially be smaller according to the trend, but afterward, the tumor started to grow to 5.6cm^3^. The mixed shrinking and growing effect of the tumor may have reflected the phenomenon of unchanged tumor size. In other periods, the treatment effect is all or none. Hence, we can see the size change relating to the CD4 count.

## Conclusion

EBV-SMT is a rare tumor, and it is important to conduct further studies when smooth muscle tumors are detected in an unusual location. In patients with HIV-related EBV-SMT, treatment should include ART. When tumor progression is noted, ART should be continued despite the presence of IRIS.

## Data Availability Statement

The raw data supporting the conclusions of this article will be made available by the authors, without undue reservation.

## Ethics Statement

The study was conducted in accordance with the Declaration of Helsinki, and written informed consent was obtained from the patient for the publication of this case report and the accompanying images.

## Author Contributions

YBC, P-LL, and C-HC contributed to conception and design of the study. Y-CM, H-LY organized the database. Y-CM, H-LY performed the statistical analysis. YBC wrote the first draft of the manuscript. YBC, P-LL, C-HC, Y-CM wrote sections of the manuscript. All authors contributed to manuscript revision, read, and approved the submitted version.

## Conflict of Interest

The authors declare that the research was conducted in the absence of any commercial or financial relationships that could be construed as a potential conflict of interest.

## Publisher’s Note

All claims expressed in this article are solely those of the authors and do not necessarily represent those of their affiliated organizations, or those of the publisher, the editors and the reviewers. Any product that may be evaluated in this article, or claim that may be made by its manufacturer, is not guaranteed or endorsed by the publisher.
